# Transcriptomic study of gastrointestinal stromal tumors with liver metastasis

**DOI:** 10.3389/fgene.2023.1007135

**Published:** 2023-02-23

**Authors:** Jianrong Guo, Shoucheng Feng, Hong Yu, Biyi Ou, Dan Jiang, Wei Zhuang, Chao Ding, Xiaojiang Chen, Miaoquan Zhang, Yudong Ling, Yi Zeng, Haibo Qiu

**Affiliations:** ^1^ Department of Gastric Surgery, State Key Laboratory of Oncology in South China, Collaborative Innovation Center for Cancer Medicine, Sun Yat-Sen University Cancer Center, Guangzhou, China; ^2^ Department of Pharmacy, Women and Children's Hospital, School of Medicine, Xiamen University, Xiamen, Fujian, China

**Keywords:** gastrointestinal stromal tumor, epithelial mesenchymal transition, liver metastasis, tumorigenesis, differentially expressed genes (DEG)

## Abstract

**Introduction:** GIST (gastrointestinal stromal tumor) is the most prominent mesenchymal neoplasms of the gastrointestinal tract, and liver is the most common metastasis site for GIST. The molecular mechanism leading to liver metastasis of GIST is currently unclear.

**Methods:** With the goal of revealing the underlying mechanism, we performed whole-genome gene expression profiling on 18 pairs of RNA samples comprised of GIST tissues (with liver metastasis) and corresponding non-tumor tissues. After identifying differentially expressed gene, functional annotation and signal pathway analyses were conducted. GSE13861, datasets that compare GIST (without liver metastasis) with adjacent tissues, served as a comparison.

**Results:** A total of 492 up-regulated genes and 629 down-regulated genes were identified as differentially expressed genes between liver metastasis tissues and non-tumor tissues. We characterized expression patterns of DEGs identified from our cohort and GSE13861 that show signatures of enrichment for functionality. In subsequent gene set enrichment analysis, differentially expressed genes were mainly enriched in Epithelial Mesenchymal Transition in both datasets. 493 genes were overlapped among our whole-genome gene expression profiling results and GSE13861, consisting 188 up-regulated genes and 305 down-regulated genes. By using CytoHubba plugin of Cytoscape, CDH1, CD34, KIT, PROM1, SOX9, FGF2, CD24, ALDH1A1, JAG1 and NES were identified as top ten hub genes in tumorigenesis and liver metastasis of GIST. higher expression levels of FGF2, JAG1, CD34, ALDH1A1 and the lower expression level of CDH1 were respectively associated with unfavorable overall survival. Meanwhile higher expression levels of CD34, FGF2, KIT, JAG1, ALDH1A were correlated with worse disease-free survival.

**Discussion:** The present study may help to provide candidate pathways and targets for treatment of GIST and prevention methods to liver metastasis.

## 1 Introduction

GIST (gastrointestinal stromal tumor) is the most prominent mesenchymal neoplasms of the gastrointestinal tract, and their prevalence is on the rise (Corless et al., 2011). Activating mutations in the receptor tyrosine kinase encoding genes KIT (KIT proto-oncogene, receptor tyrosine kinase) or PDGFRA (platelet -derived growth factor receptor alpha) are extensively seen in GISTs (Serrano and George, 2020). These mutations cause constitutive activation of KIT or PDGFRA-mediated ligand independent activation and signaling (Joensuu et al., 2013). GISTs can appear everywhere in the gastrointestinal tract, although they’re most prevalent in the stomach (50%–60%) and small intestine (30%–35%), with the colon and rectum (5%) and oesophagus (1%) (Joensuu et al., 2012). Liver metastasis (LM) from GIST is very common, and a primary tumor is diagnosed simultaneously in 15%–50% of cases. Furthermore, after excision of a high-risk GIST, up to 40%–80% of individuals may emerge with liver metastasis over a period of about 2 years (Ng et al., 1992; DeMatteo et al., 2000; DeMatteo et al., 2009). However, the mechanisms of GIST invasion and acquisition of the potential to metastasize are still unknown. Acquiring a better knowledge of the molecular process behind liver metastasis of GIST is crucial, as it might result in new anticancer treatment targets and greatly contribute to advances in diagnostic approaches.

Gene chip, also known as gene profile, is a gene detection method that has been used for over a decade. Gene chips can instantly identify all of the genes’ expression information within the same sample time-point, making them ideal for detecting differentially expressed genes (DEGs) (Wang, 2000). Therefore, we collected GIST tissues of patients with liver metastasis and corresponding non-tumor tissues (stomach and intestinal tissue) yielding sufficient RNA for gene expression profiling. Meanwhile we also downloaded mRNA microarray data from the Gene Expression Omnibus (GEO) and jointly analyzed our gene expression profiling data with online data for identifying differentially expressed genes which may play an important role in tumorigenesis and liver metastasis of GIST. Gene Ontology (GO) annotation and Kyoto Encyclopedia of Genes and Genomes (KEGG) pathway enrichment analyses were applied to further provide an overview of the function of the screened DEGs. Then a protein-protein interaction (PPI) network was constructed to determine the hub genes and survival analyses of the screened hub genes were carried out using Gene Expression Profiling Interactive Analysis (GEPIA).

In this study, we first performed gene chip detection on GIST tumor sample and peri cancerous tissues of 18 GIST patients with liver metastases, obtained microarray dataset, and obtained organized microarray dataset of GIST with no liver metastasis and paracancer tissues from the GEO database. Differentially expressed genes were analyzed separately, and the enrichment of DEGs in the two datasets were analyzed. The STRING website and Cytoscape software were used to find out the key genes that promote the tumorigenesis and liver metastasis of GIST. Finally, we explored the potential of these key genes as prognostic markers of gastrointestinal tumors using Kaplan–Meier Survival analyses. This study helps us better understand the molecular mechanism of GSIT tumorigenesis and liver metastasis.

## 2 Materials and methods

### 2.1 Clinical samples

GIST tissues of patients with liver metastasis and corresponding non-tumor tissue (stomach and intestinal tissue) samples were obtained from Sun Yat-sen University Cancer Center under protocols approved by the institutional review board at Sun Yat-sen University Cancer Center. Written informed consent was obtained from all patients enrolled in the study. All experiments using clinical samples were carried out in accordance with the approved guidelines.

### 2.2 Microarray analysis

All samples were frozen in liquid nitrogen at −80°C. The total RNA of samples was extracted by TRIZOL method, and the total RNA was examined by NanoDrop 2000 and Agilent Bioanalyzer 2100. The qualified sample goes into the chip experiment. The standards of quality control are: Thermo NanoDrop 2000:1.7 < A260/A280 < 2.2; Agilent 2100 Bioanalyzer: RIN ≥ 7.0 and 28S/18S > 0.7. Affymetrix GeneChip Human Primeview array (Affymetrix, Santa Clara, CA, United States) was used to analyze global expression pattern of 28,869 well-annotated genes. RNA samples were amplified and labeled using the 3′IVT Expression Kit and GeneChip WT Terminal Labeling and Control Kit from Affymetrix. Affymetrix’s GeneChip Fluidics Station 450 was used to carry out the normal washing treatment after the samples were hybridized at 45°C for 16 h. The arrays were then scanned using the GeneChip Scanner 7G procedure. Quantile normalization of gene expression was performed using the normalizeBetweenArrays function in limma.

We also downloaded the following gene expression profiles from the GEO: GSE13861 (including six GIST and 19 surrounding normal fresh frozen tissues) (Cho et al., 2011) for further analysis.

### 2.3 DEG identification

R language limma package was used to identify DEGs in our cohort and GSE13861 separately. The log-fold change (FC) in expression and adjusted *p*-values (adj. P) were determined. The adj. P using the Benjamini–Hochberg method with default values were applied to correct the potential false-positive results. DEGs were defined as genes that satisfied the specified cutoff criterion of adj. *p* > 0.05 and | logFC | > 2.0. The Venn diagram online tool was used to look at the intersecting genes. In order to illustrate the volcano plot of DEGs, visual hierarchical cluster analysis was also carried out.

### 2.4 GO annotation and KEGG pathway enrichment analyses of DEGs

To reveal the functions of DEGs, GO annotation and KEGG pathway enrichment analyses were conducted. Biological process (BP), cellular component (CC), and molecular function (MF) were the three categories that made up the GO terms. Statistical significance was determined to be adj. *p* < 0.05. Resulting *p*-values are adjusted for multiple testing using the “Benjamini–Hochberg” method.

### 2.5 Gene set enrichment analysis (GSEA)

To find out the different mechanisms between GIST with liver metastasis and GIST without metastasis, GSEA (Version: 3.0; http://software.broadinstitute.org/gsea/index.jsp) was performed (Subramanian et al., 2005). The threshold was set at *p* < 0.05.

### 2.6 Construction of PPI network and screening of hub genes

A database called Search Tool for the Retrieval of Interacting Genes (STRING) is used to study the functional protein association networks (Szklarczyk et al., 2017). The filtered DEGs had already been added to the STRING database. All PPI pairs with a cumulative score greater than 0.4 were retrieved. High-degree nodes seem to be essential for maintaining the network’s overall stability. The degree of all nodes was calculated by Cytoscape (v3.6.1) plugin cytoHubba using the MCC algorithm (Chin et al., 2014), in this experiment, the genes with the top 10 highest MCC score values were considered as hub genes.

### 2.7 Kaplan–meier survival analyses of the hub genes

Survival analysis of hub genes was based on Kaplan–Meier Survival analyses, using GEPIA (http://gepia.cancer-pku.cn/) tool. According to the expression of each hub gene, the cancer patients were divided into low or high expression group based on the median mRNA expression of hub genes, at statistical significance of *p* < 0.05.

## 3 Result

### 3.1 Characteristics of GIST patients with liver metastasis in our cohort

Our cohort consisting of 18 paired GIST tissues of patients with liver metastasis (LM) and corresponding non-tumor tissue (NT) samples. Details of mutations, clinical features for the 18 GIST patients with liver metastasis are presented in Table 1. Eight of the 18 patients were male and 10 were female. The youngest patient was 23 and the oldest was 71. Four of the 18 GISTs are small-intestine GISTs, and the remaining 14 are stomach GISTs. All patients presented with liver metastases. And all of the patients harbored a single non-synonymous mutation in KIT (Kit exon 11). The tumor size, mitotic index and location of primary tumors are demonstrated in Table 1.

**TABLE 1:** Details of mutations, clinical features for the 18 GIST patients with liver metastasis.

No.	Age	Sex	Site	Size (cm)	Mitotic index	Grade	Metastasis	Mutation
1	48	M	small intestine	4	200	high	liver	K11
2	71	F	stomach	7.8	50	high	liver	K11
3	58	M	small intestine	6	90	high	liver	K11
4	63	F	stomach	10.3	10	high	liver	K11
5	59	M	small intestine	8	55	high	liver	K11
6	57	M	stomach	7	>5	high	liver	K11
7	23	M	stomach	4.3	15	high	liver	K11
8	54	F	small intestine	4.7	4	low	liver	K11
9	57	F	stomach	3.9	>30	high	liver	K11
10	50	F	stomach	2.5	6	medium	liver	K11
11	60	M	stomach	3.7	6	medium	liver	K11
12	48	F	stomach	4.5	15	high	liver	K11
13	33	F	stomach	6	4	medium	liver	K11
14	57	F	stomach	2.3	<3	low	liver	K11
15	59	F	stomach	4.9	14	high	liver	K11
16	59	M	stomach	5	9	medium	liver	K11
17	68	F	stomach	2.6	20	high	liver	K11
18	52	M	stomach	7.5	>10	high	liver	K11

### 3.2 Identification of differentially expressed genes (DEGs)

**FIGURE 1:**
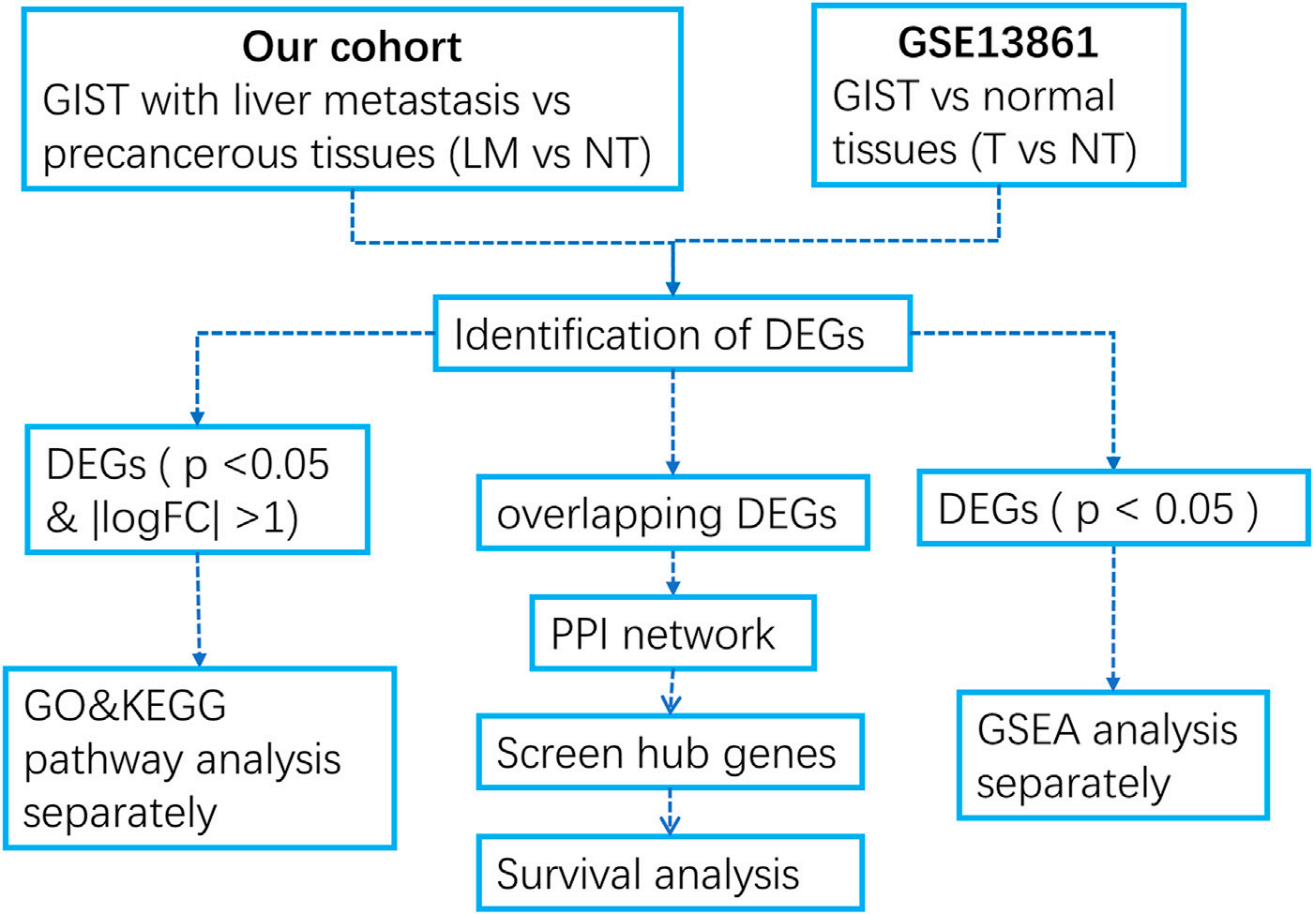
Flow diagram of the data collection and method implementation in this work.

**FIGURE 2:**
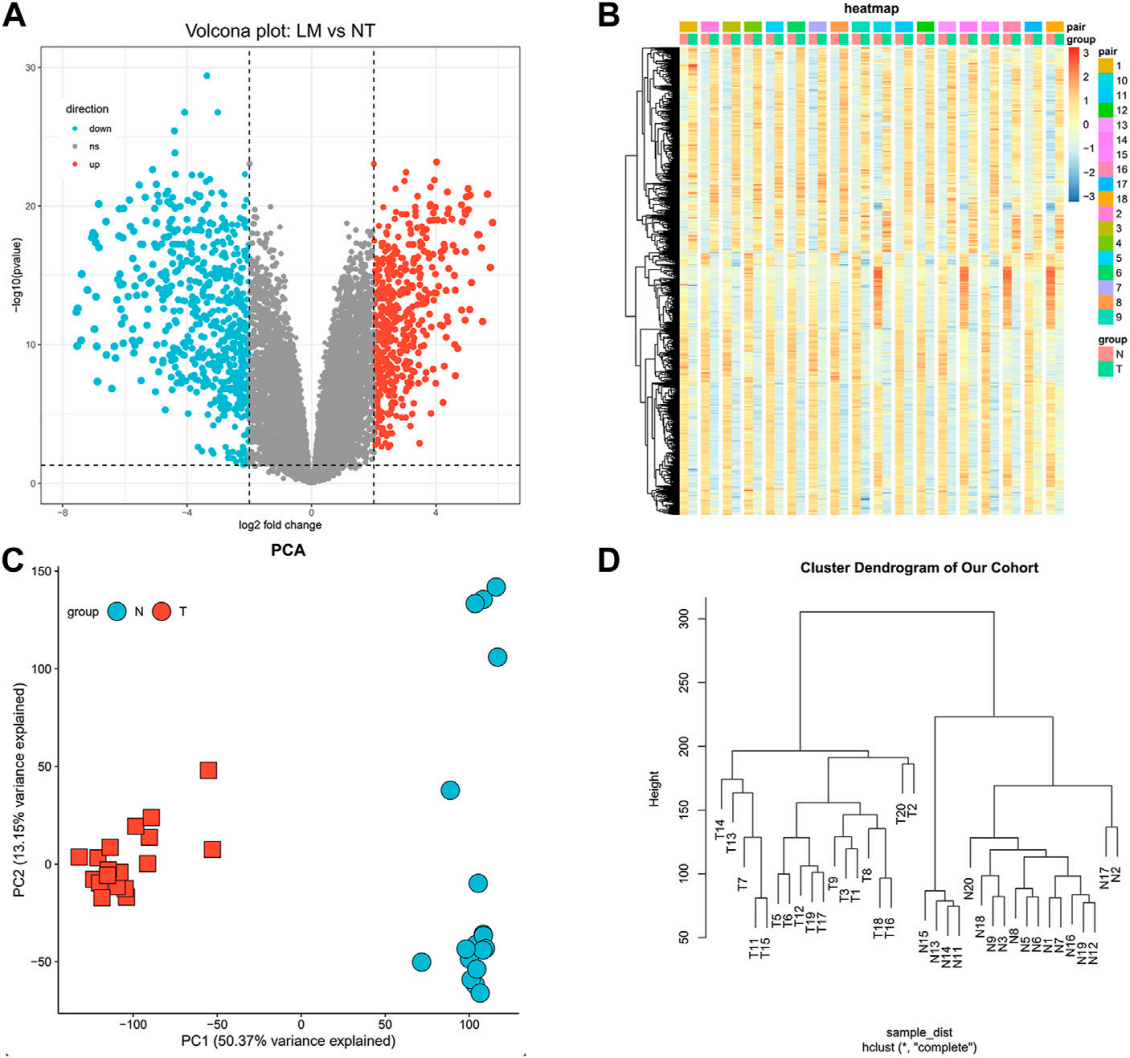
Identification of differentially expressed genes. **(A)** Volcano map of differentially expressed genes (Upregulated genes in red, downregulated genes in blue). **(B)** Hierarchical clustering heatmap of DEGs screened on the basis of FC > 2.0 and a corrected *p*-value < 0.05. **(C)** Shows PCA results of our cohort. **(D)** Visual hierarchical cluster analysis.

We developed a flow diagram to show our process (Figure 1). To characterize the tumor biology of GIST with liver metastasis, we performed whole-genome gene expression profiling in 18 pairs of RNA samples comprised of GIST with LM and NT tissues. 1121 genes were found to differentially express between LM and adjacent tissues, including 492 upregulated genes and 629 downregulated genes (Supplementary Data Sheet S1). Volcano map of DEGs was shown in Figure 2A. Subsequently, heatmap of DEGs was created, in which the mRNA expression profiles of LM and NT resulted in obviously separate clusters (Figure 2B). Principle Component Analysis (PCA) and hierarchical cluster analysis results were demonstrated in Figures 2C, D. GSE13861 (including 6 GIST and 19 surrounding normal fresh frozen tissues) is a dataset that compare GIST without liver metastasis with adjacent tissues, which serves as a comparison. DEGs in GSE13861 were calculated according to the criteria of *p* < 0.05 and |logFC|>2.0. 924 genes were found to differentially express between GIST and adjacent tissues, including 313 upregulated genes and 611 downregulated genes (Supplementary Data Sheet S2). Volcano map of DEGs is shown in Supplementary Figure S1A. Hierarchical clustering heatmap of DEGs was shown in Supplementary Figures S1B, C Shows PCA results of GSE13861. Hierarchical cluster analysis was visualized and important details were demonstrated in Supplementary Figure S1D.

### 3.3 GO and KEGG analysis of DEGs reveal the different enrich patterns of GIST with LM and GIST without LM

**FIGURE 3:**
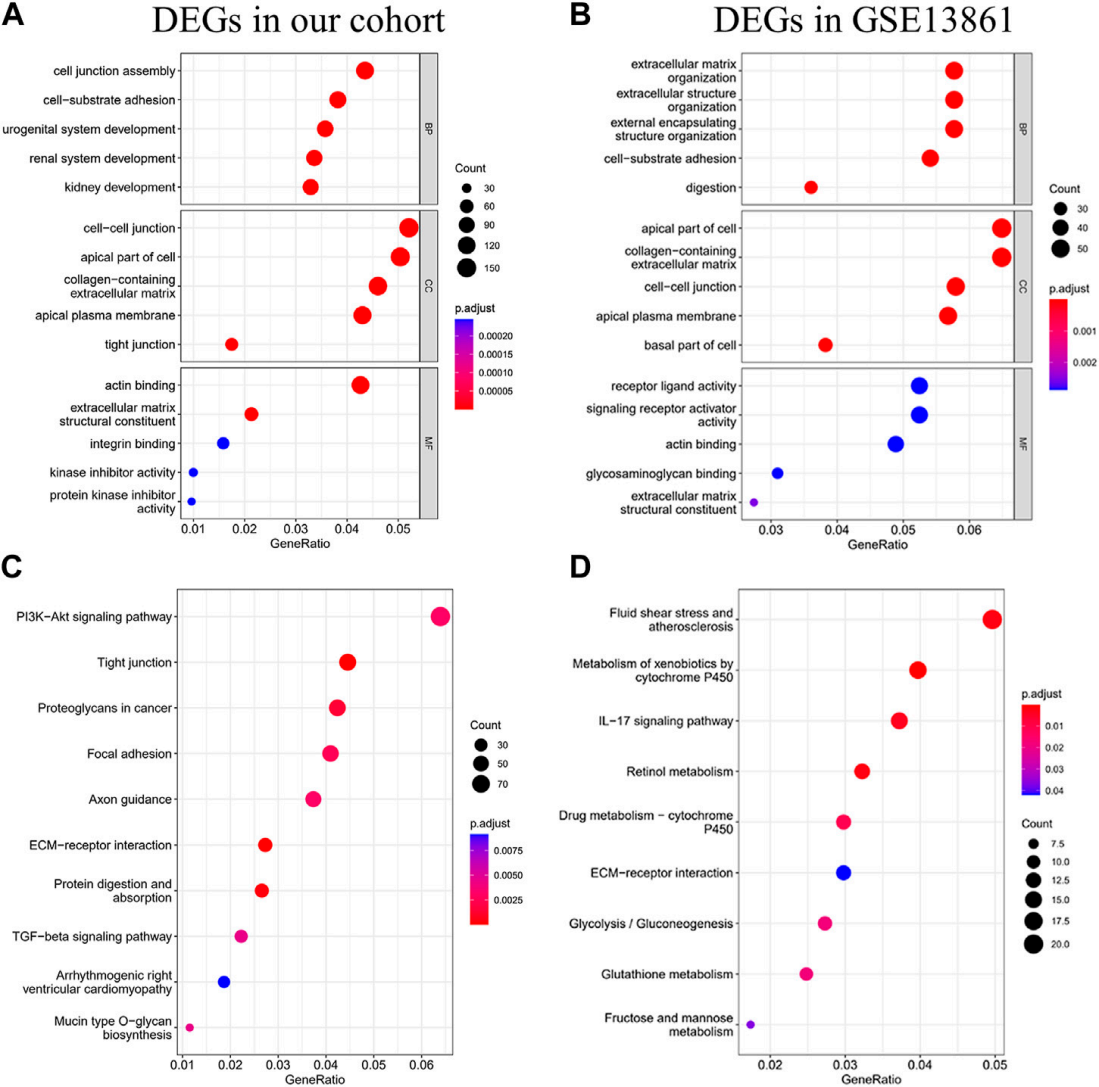
GO and KEGG analysis of DEGs. GO analysis **(A)**, and KEGG analysis **(C)** of DEGs in our cohort. GO analysis **(B)**, and KEGG analysis **(D)** of DEGs in GSE13861 dataset.

**FIGURE 4:**
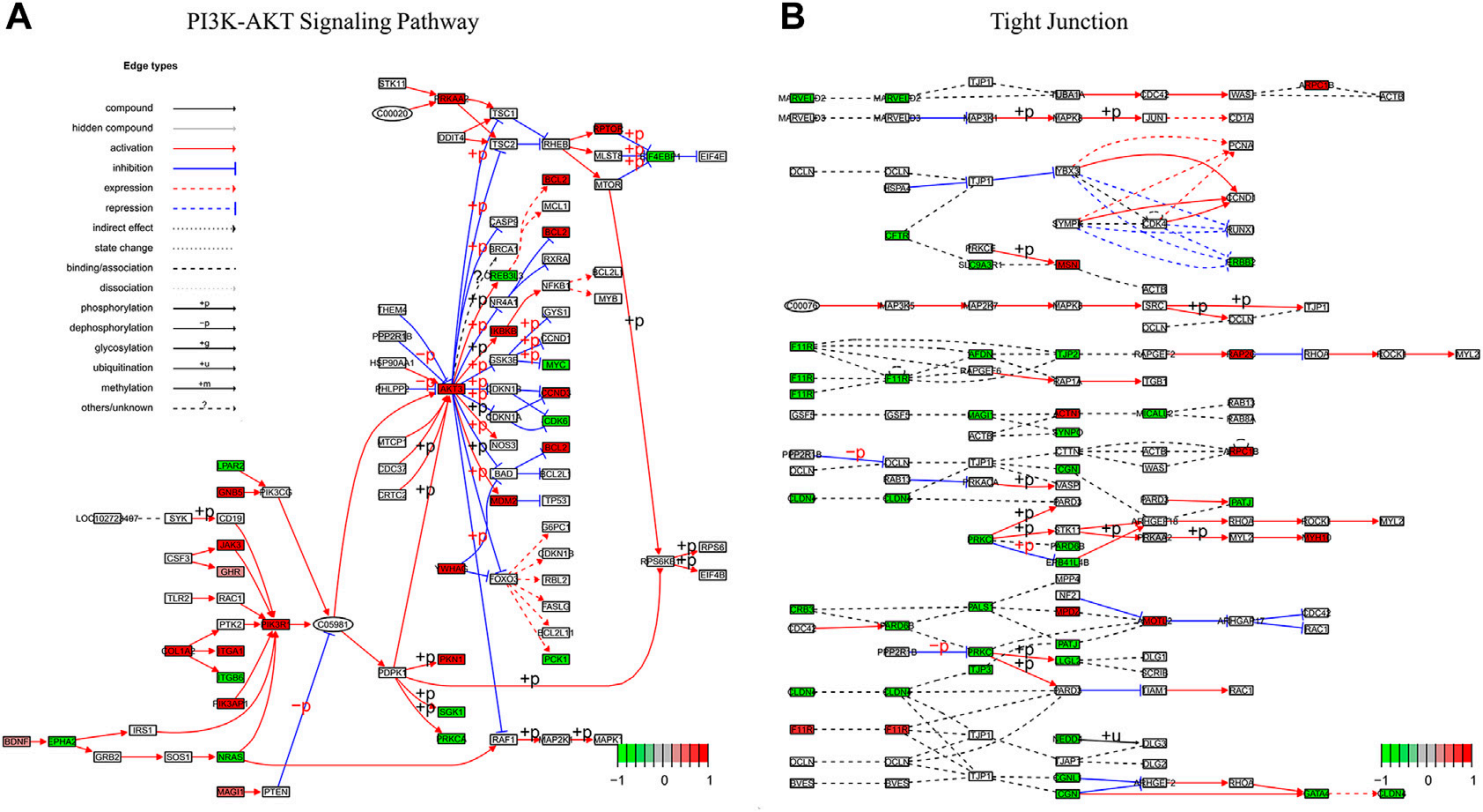
Pathview map of **(A)** PI3K-AKT Signaling Pathway (map 04,151) and **(B)** Tight Junction (map04530) using data of our cohort. Upregulated genes in red, downregulated genes in green.

To characterize the biological mechanism of GIST liver metastasis, gene enrichment analysis including Gene ontology (GO) and Kyoto Encyclopedia of Genes and Genomes (KEGG) pathway enrichment analyses were conducted. DEGs acquired from the two datasets were subjected to enrichment separately. For GO biological process (BP), DEGs in our cohort were mainly enriched in *cell junction assembly*, *cell-substrate adhesion* and *urogenital development*, while DEGs in GSE13861 were mainly enriched in *extracellular matrix organization*, *extracellular structure organization* and *external encapsulating structure organization*. In terms of cellular component (CC), DEGs in our cohort were mainly enriched in *collagen-containing extracellular matrix*, *cell-cell junction* and *apical part of cell*. The CC enrichment results of GSE13861 were very similar to our cohort. For GO molecular function (MF), results were also similar between these two cohorts (Figures 3A, B). We further explored the function significance of these DEGs using KEGG pathway analysis. DEGs in our cohort were mainly enriched in *PI3K-Akt signaling pathway* and *Tight junction*, while DEGs in GSE13861 were mainly enriched in *Fluid shear stress and atherosclerosis* and *Metabolism of xenobiotics by cytochrome P450* (Figures 3C, D). Changes in gene expression in *PI3K-Akt signaling pathway* and *Tight junction* signaling pathways in our cohort are depicted in detail in Figures 4A, B.

### 3.4 Gene set enrichment analysis reveal the differences between GIST with LM and GIST without LM

**FIGURE 5:**
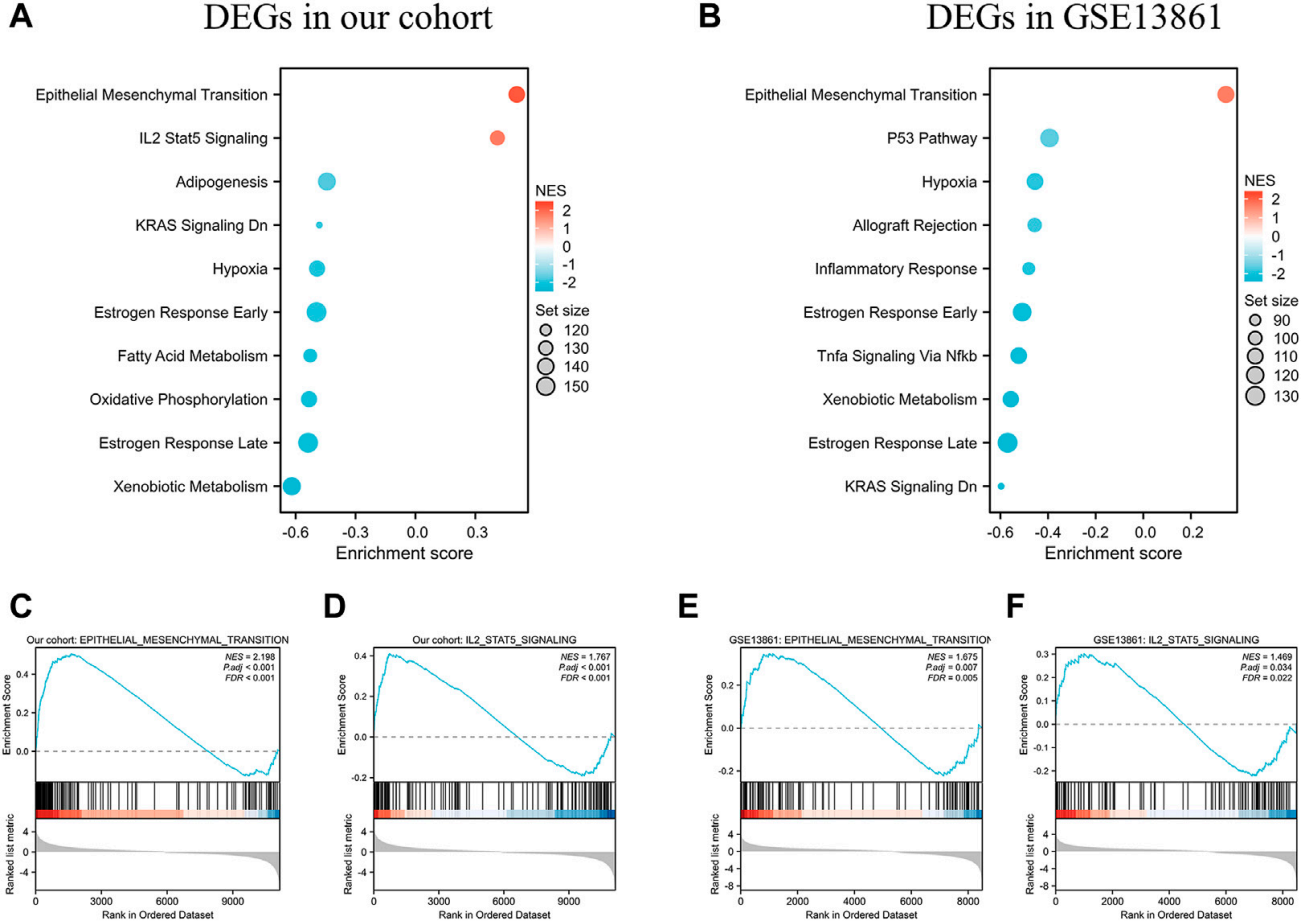
GSEA analysis of DEGs in the data sets. **(A)** The top 10 enriched KEGG items for the DEGs in our cohort, and **(B)** DEGs in GSE13861 dataset. Taking *p* < 0.05 as the boundary value. Significant enrichment of the Epithelial Mesenchymal Transition **(C)** and IL-2 STAT5 Signaling **(D)** with DEGs in our cohort. Significant enrichment of the Epithelial Mesenchymal Transition **(E)** and IL-2 STAT5 Signaling **(F)** with DEGs in GSE13861.

GSEA was performed to identify the gene sets that were statistically different between the normal controls and GIST group (Taking *p* < 0.05 as the boundary value). The results illustrated that *Epithelial mesenchymal transition* (EMT) was the most significantly upregulated pathway in both cohorts (Figures 5A–C, E). DEGs in our cohort were also positively correlated and significantly enriched in *IL2 Stat5 Signaling* (Figures 5A, D, NES = 1.767 & P.adj <0.001). While in GSE13861, *IL2 Stat5 Signaling* was not in the top10-enriched pathways (Figure 5B).

### 3.5 PPI network construction and hub genes selection and analysis

**FIGURE 6:**
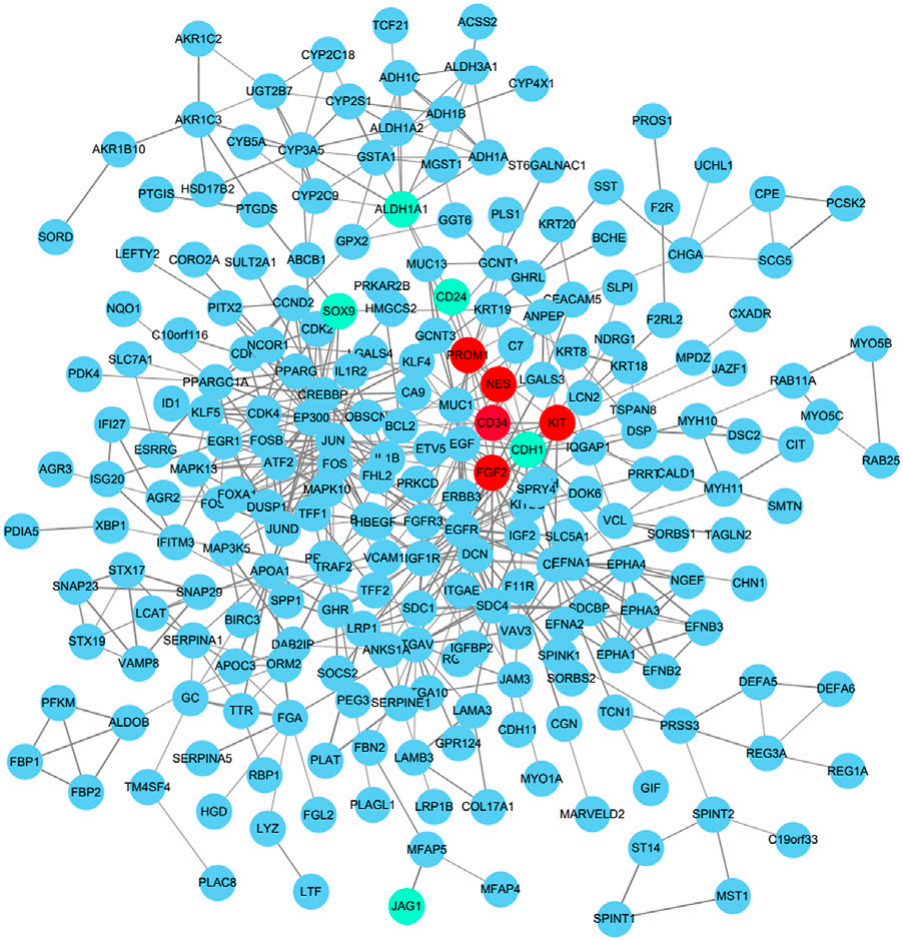
Protein–protein interaction network of 188 upregulated genes and 305 downregulated genes were analyzed using Cytoscape software. The edges between 2 nodes represent the gene-gene interactions. Upregulated hub genes in red, downregulated hub genes in teal.

**FIGURE 7:**
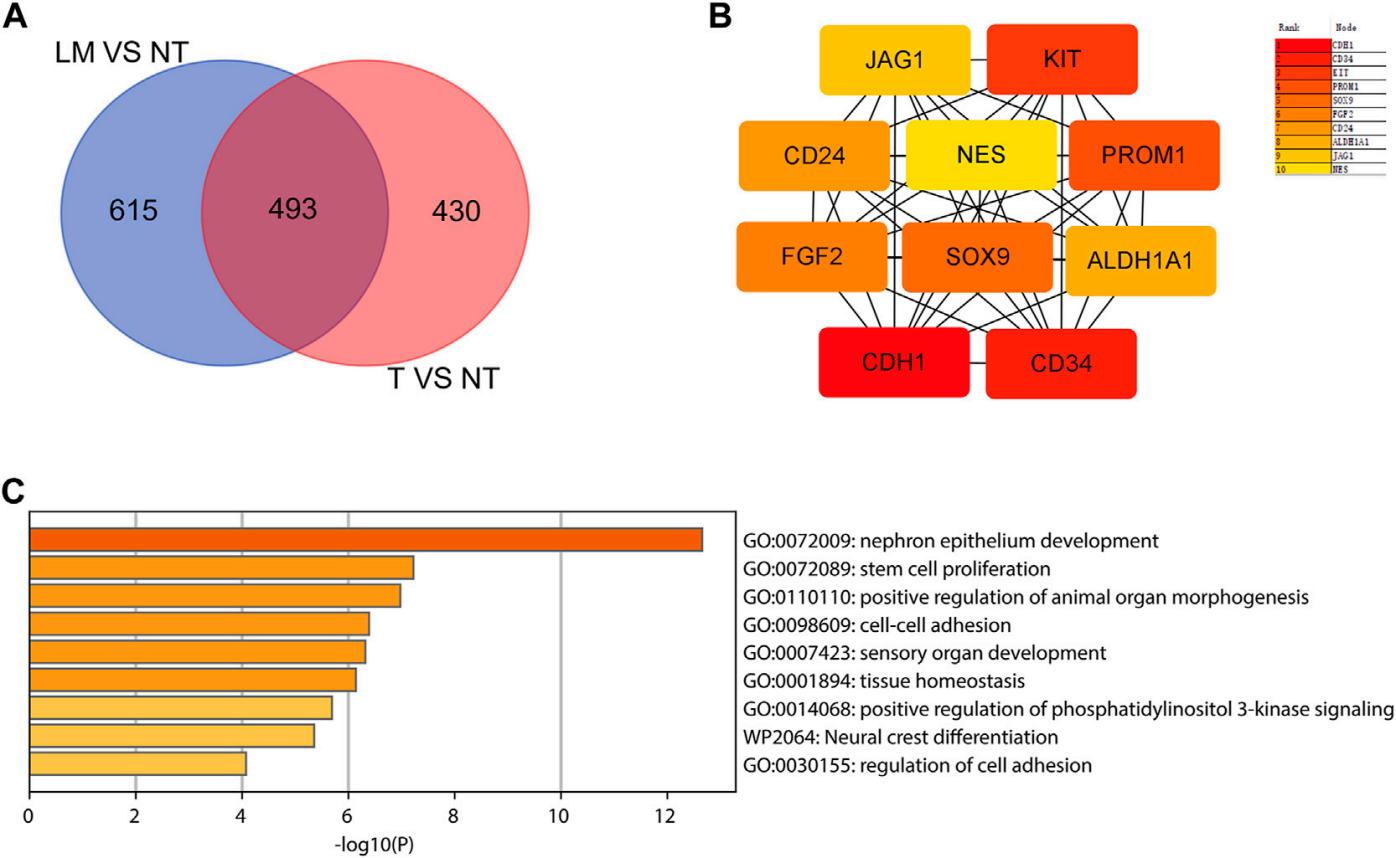
Hub genes selection and analysis. **(A)** Venn diagram shows the 493 overlapping DEGs. **(B)** The top 10 hub genes in the PPI network were screened by Cytoscape plugin cytoHubba. The 10 identified hub genes such as CDH1, CD34, KIT, PROM1, SOX9, FGF2, CD24, ALDH1A1, JAG1, NES are displayed from red (high degree value) to yellow (low degree value). **(C)** GO and KEGG pathway enrichment analysis of the 10 hub genes.

To identify those genes which play significant roles in both tumorigenesis and liver metastasis of GIST, GSE13861 dataset containing GIST primary tumor tissues (PT) and corresponding non-tumor tissues (NT) was co-analyzed. The Venn diagram (Figure 7A) illustrated a total of 493 genes overlapped among our microarray results and GSE13861, consisting 188 upregulated genes and 305 downregulated genes (Supplementary Data Sheet S3). Using the STRING and Cytoscape databases, a PPI network of potential interactions between overlapping genes was constructed (Figure 6). The hub genes were selected from the PPI network using the MCC algorithm of CytoHubba plugin. According to the MCC scores, the top ten highest-scored genes included CDH1, CD34, KIT, PROM1, SOX9, FGF2, CD24, ALDH1A1, JAG1, and NES (Figure 7B and Supplementary Data Sheet S4). The abbreviations, names, and functions of these genes are displayed in Table 2. The function of these hub genes was analyzed by Metascape, in which as expected, these genes were mainly enriched in pathways in cell-cell adhesion (Figure 7C).

**TABLE 2:** Details of hub genes.

Gene symbol	Degree	Full name	Gene function
CDH1	63	Epithelial cadherin	Loss of CDH1 is thought to contribute to progression in cancer by increasing proliferation, invasion, and/or metastasis
CD34	40	CD34	CD34 is a cell surface glycoprotein and function as a cell-cell adhesion factor.
KIT	39	KIT proto-oncogene receptor tyrosine kinase	Mutations in this gene are associated with gastrointestinal stromal tumors, mast cell disease, acute myelogenous leukemia, and piebaldism.
PROM1	37	prominin-1	PROM1 is often expressed on adult stem cells, where it is thought to function in maintaining stem cell properties by suppressing differentiation.
SOX9	37	SRY-box transcription factor 9	SOX-9 plays a pivotal role in male sexual development; by working with Sf1, SOX-9 can produce AMH in Sertoli cells to inhibit the creation of a female reproductive system.
FGF2	36	fibroblast growth factor 2	FGF2 is involved in a variety of biological processes, including cell growth, morphogenesis, tissue repair, tumor growth and invasion.
CD24	34	CD24	CD24 is overexpressed in many cancers and some cancer stem cells and is associated with the development, invasion, and metastasis of cancer cells.
ALDH1A1	32	aldehyde dehydrogenase 1 family member A1	High ALDH1A1 activity is closely related to stemness phenotype of several tumors, possibly contributing to cancer progression and diffusion in the body.
JAG1	31	jagged canonical Notch ligand 1	JAG1/Notch signaling cascades activate a number of oncogenic factors that regulate cellular functions such as proliferation, metastasis, drug-resistance, and angiogenesis.
NES	30	Nestin	Nestin may be a marker for newly synthesized tumor vessels and a therapeutic target for tumor angiogenesis.

### 3.6 Validation and prognostic value of hub genes

**FIGURE 8:**
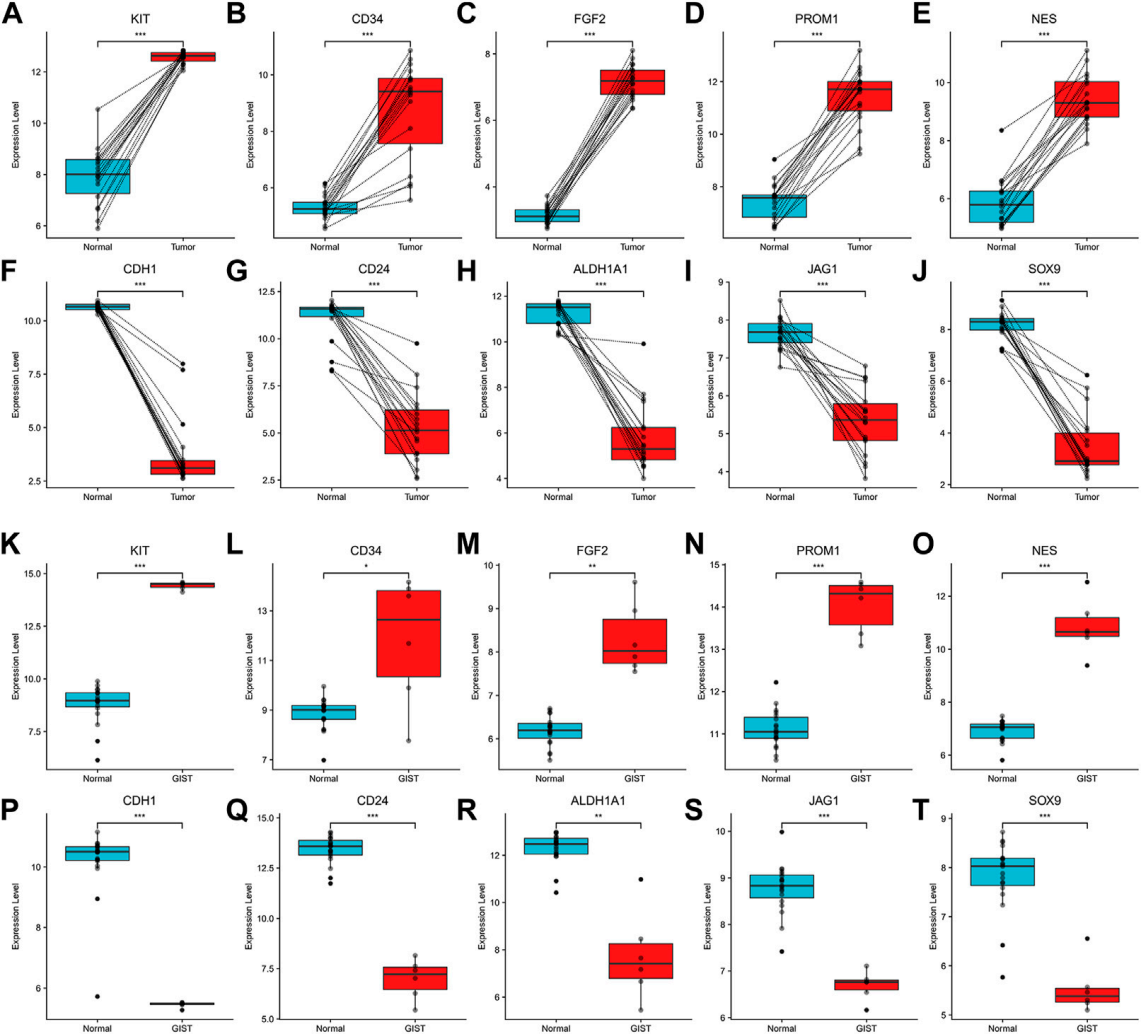
Expression of hub genes. **(A–J)**. Expression of KIT, CD34, FGF2, PROM1, NES, CDH1, CD24, ALDH1A1, JAG1 and SOX9 in Our cohort and **(K–T)** GSE13861. *p < 0.05, **p < 0.01, ***p < 0.001.

**FIGURE 9:**
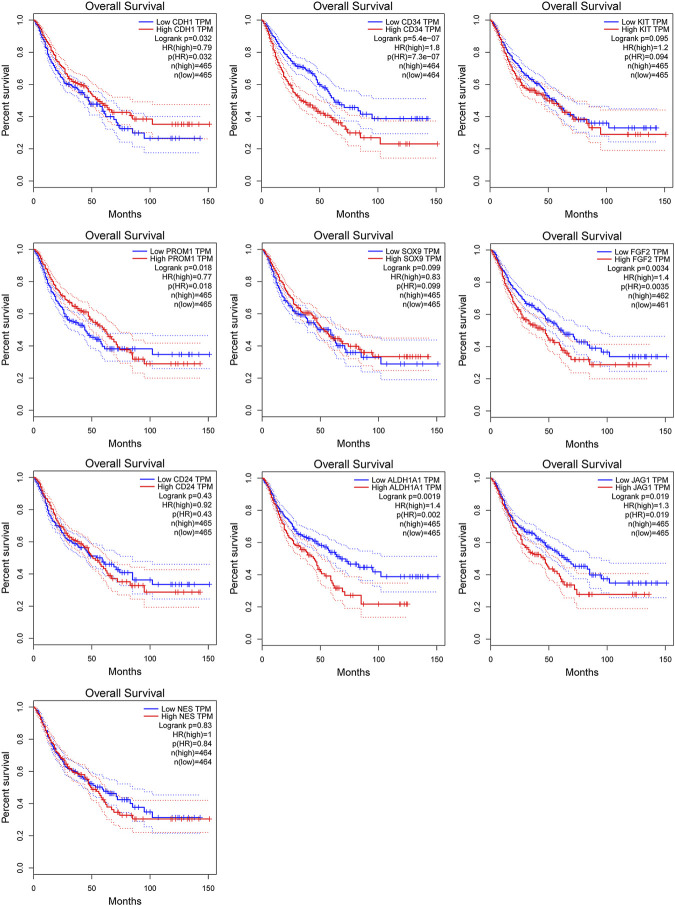
Kaplan-Meier curves of hub genes expression and overall survival in gastrointestinal tumors. Data are presented as the hazard ratio with a 95% confidence interval. Log-rank *p* < 0.05 was regarded as statistically significant.

**FIGURE 10:**
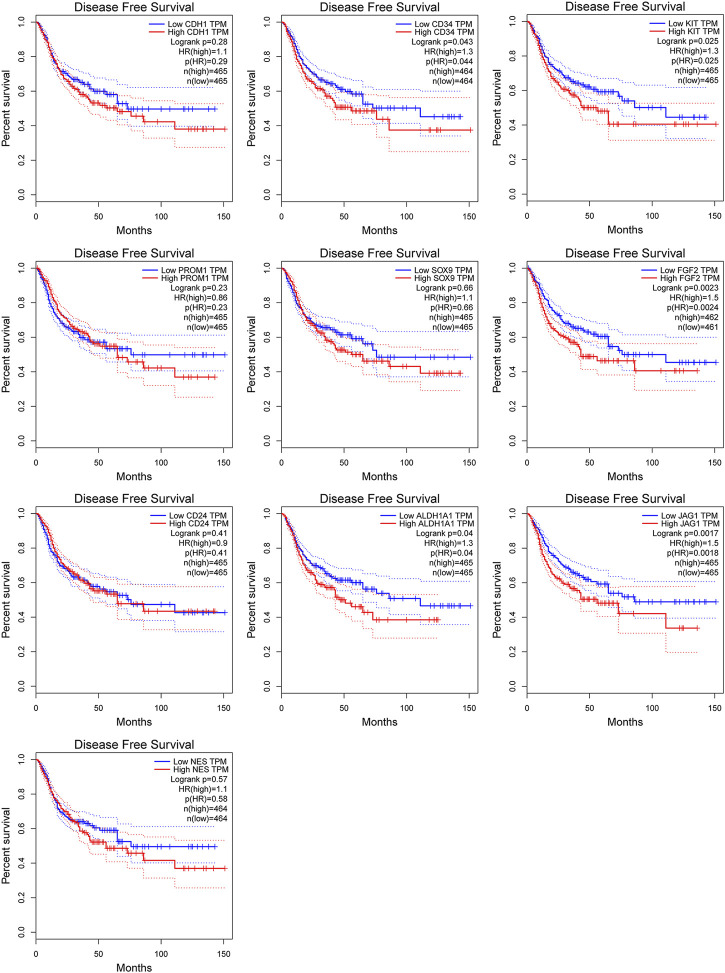
Kaplan-Meier curves of hub genes expression and disease-free survival in gastrointestinal tumors. Data are presented as the hazard ratio with a 95% confidence interval. Log-rank *p* < 0.05 was regarded as statistically significant.

Among above mentioned 10 hub genes, the expressions of CD34, KIT, PROM1, NES, and FGF2 respectively were higher in GIST (with LM) tissues (Figures 8A–E) compared to NT tissues (*p*-values all <0.001). Meanwhile reverse trend was found for the expressions of the rest hub genes CDH1, SOX9, CD24, ALDH1A1, and JAG1 (Figures 8E–J, *p*-values all <0.001). These results are nearly identical to the findings from the GES13861 dataset (Figures 8K–T). Prognostic significance of hub genes was investigated in several types of gastrointestinal tumors including stomach adenocarcinoma, colon adenocarcinoma, esophageal carcinoma and rectal adenocarcinoma by the GEPIA database. The Kaplan-Meier analyses suggested that higher expression levels of FGF2, JAG1, CD34, and ALDH1A1 and the lower expression level of CDH1 were respectively associated with worse overall survival (OS) (Figure 9). Meanwhile higher expression levels of CD34, FGF2, KIT, JAG1, and ALDH1A were correlated with worse disease-free survival (DFS) (Figure 10).

## 4 Discussion

During the past decade, GIST has become the prominent focus of molecularly targeted therapy for solid tumors (Poveda et al., 2017; Hemming et al., 2018). GIST are more prevalent than previously thought, according to population-based studies (Corless and Heinrich, 2008). The incidence of GIST was found to be 14.5 per million population, with the highest frequency being observed in older individuals and there was no gender difference (Gold and DeMatteo, 2006). The hallmarks of cancer consist of six biological traits: sustaining proliferative signaling, evading growth suppressors, evasion of apoptosis, limitless replicative potential, inducing angiogenesis, and ability to invade and metastasize (Hanahan and Weinberg, 2011). It is worth noting that the last characteristic, invasion and metastasis is vital for progressive nature of cancer. Many malignancies favor certain organs as metastatic sites, including the lungs, bone marrow, and liver. Liver metastases are a major cause of death in patients with colorectal cancer. The liver environment, which includes ECM and stromal cells, may encourage metastatic colonization. Metastatic colorectal cancer cell lines responded more favorably to ECM derived from primary rat hepatocytes than to ECM from fetal rat fibroblast cultures (Zvibel et al., 1998). The D6.1A tetraspanin, a cell-surface organizer, interacted with the 64 integrin and enhanced liver colonization by pancreatic cancer cells injected intraperitoneally (Herlevsen et al., 2003).

Patients with GIST have a high risk of recurrence (about 55–72 percent) and a dismal survival rate due to malignant cells preferentially metastasizing to liver tissue (DeMatteo et al., 2000; Bayraktar et al., 2010). Cho et al. discovered that Compared to KIT mutation-negative GISTs, KIT mutation-positive GISTs had more frequent liver metastases and worse mortality (Cho et al., 2006). Wang et al. reported that the KIT exon 11,557-558 deletion upregulates CXCR4 by increasing ETV1 binding to the CXCR4 promoter in GIST cells, which in turn encourages liver metastasis (Wang et al., 2016). As such, to better understand GIST biological behavior and inform the development of treatment strategies, it is critical to identify the significant genes that regulate the liver metastasis of GIST. Advances in bioinformatics have been conducive to identify molecular targets that indicate the progression of GIST (Amirnasr et al., 2019; Ohshima et al., 2019).

In this study, a total of 492 upregulated genes and 629 downregulated genes were identified in GIST with LM compared to corresponding NT. Function annotation based on GO and KEGG analyses demonstrated that DEGs were mainly enriched in *cell junction assembly*, *tight junction*, *actin binding* and *PI3K-Akt signaling pathway*. GSEA results indicated that *IL-2 STAT5 Signaling* may be a vital pathway which promotes liver metastasis of GIST. Meanwhile, *EMT signal pathway* is the most significant and positive enriched pathway in both our cohort and GSE13861, which indicated that EMT may play a significant role in tumorigenesis and liver metastasis of GIST. Furthermore, to identify genes which play essential roles in both tumorigenesis and liver metastasis of GIST, our data and GSE13861 dataset were co-analyzed. A totally of 493 genes overlapped among our microarray results and GSE13861, including 188 upregulated genes and 305 downregulated genes. Then a PPI network of putative interactions between overlapping genes was created using the STRING and Cytoscape databases and hub genes were selected from the PPI network using the MCC algorithm of CytoHubba plugin. According to the MCC scores, the top ten highest-scored genes were CDH1, CD34, KIT, PROM1, SOX9, FGF2, CD24, ALDH1A1, JAG1, and NES.

The phosphatidylinositol PI3K/AKT/mTOR pathway is a critical survival pathway for cell proliferation, apoptosis, autophagy and translation in neoplasms (Patel, 2013). Constitutive autophosphorylation of RTKs has an impact on the activation of the PI3K/AKT/mTOR pathway (Vara et al., 2004; Fruman and Rommel, 2014). In several preclinical and early-stage clinical trials PI3K/AKT/mTOR signaling inhibition has been considered as a promising targeted therapy strategy for GISTs (Duan et al., 2020). Our results suggest that, unlike GIST, liver-metastatic GIST has more genes enriched in the *PI3K-Akt signaling pathway*. We hypothesized that *PI3K-Akt signaling pathway* is an important pathway to promote liver metastasis of GIST. It can be used as a target to prevent and treat liver metastasis of GIST.

Tight junction is the most talked-about structure in epithelial and endothelial cells because they control permeability (Jiang et al., 1999; Tsukita et al., 1999). It is an area where neighboring cells’ plasma membranes make a sequence of connections that appear to totally obstruct the extracellular space, forming an intercellular barrier and intramembrane diffusion fence (Wong and Gumbiner, 1997). The majority of malignancies are characterized by abnormal growth control, tissue architecture loss, and loss of differentiation. The feature that cancer cells’ mutual adhesiveness is much less than that of normal cells is a key characteristic of cancer cells (Martin and Jiang, 2009). Reduced cell-cell interaction leads cancer cells to rebel against the social order, resulting in the breakdown of overall tissue architecture, a morphological hallmark of malignancy. The loss cell-cell junction and tight junction are changes associated with cancer progression to an invasive, metastatic state (Thomson et al., 2011).

The cytokine interleukin-2 (IL-2) was first discovered in 1976 as a T cell growth factor (Morgan et al., 1976). While IL-2 has been shown to activate several STAT family members, including STAT1, STAT3, and STAT5, STAT5 is the predominant IL-2 signaling molecule (Hou et al., 1995; Lin et al., 1995). Indeed, IL-2 has also been shown to signal *via* the Mitogen Activated Protein Kinase (MAPK) pathway, *via* extracellular signal-regulated kinase (ERK), as well as the PI3K pathway (González-García et al., 1997; Liao et al., 2013; Ross and Cantrell, 2018). In this study, we identified *IL-2 STAT5 Signaling* is the second and positively enriched pathway using GSEA in DEGs in our cohort, while in GSE13861, *IL-2 Stat5 Signaling* was not in the top10-enriched pathways. This result indicates that *IL-2 STAT5 Signaling* may be a vital pathway which promotes liver metastasis of GIST.

The extracellular matrix (ECM) performs many functions in addition to its structural role; as a major component of the cellular microenvironment it influences cell behaviors such as proliferation, adhesion and migration, and regulates cell differentiation and death (Hynes, 2009). Abnormal ECM dynamics can result in uncontrolled cell proliferation and invasion, failure of cell death, and loss of cell differentiation, which can lead in congenital abnormalities and pathological processes such as tissue fibrosis and cancer. As the ECM’s significance in tumor progression becomes more evident, cancer treatment strategies have started to focus on specific ECM components in an effort to reduce metastasis (Walker et al., 2018; Paolillo and Schinelli, 2019; Girigoswami et al., 2021).

Epithelial mesenchymal transition (EMT) is a crucial developmental process that triggers the transdifferentiation of polarized epithelial cells into mesenchymal cells during tumor invasion and metastasis (Kalluri and Weinberg, 2009; Polyak and Weinberg, 2009). Cancer cells acquire invasive and metastatic characteristics with activation of EMT, which facilitates effective colonization of distal target organs (Tsai and Yang, 2013). In line with previous study, we found that EMT signal pathway enriched in GIST tissues of patients with liver metastasis compared to corresponding pericancerous tissues, which indicated that EMT may play a significant role in liver metastasis of GIST.

E-cadherin (also known as cadherin-1 or CDH1), a protein belonging to the cadherin family, is possibly one of the most potent and extensively researched regulators of adhesion. Together with associated Catenins, E-cadherin is essential for regulating cell adhesion, signaling and transcription in cancers and controlling metastatic progression (Jiang and Mansel, 2000). Alteration in cell adhesion molecules (CAMs), such as E-cadherin affect the processes of cell-cell adhesion and cell-matrix adhesion and subsequently their metastatic potential. It also regulates the cell cycle regulators p27kip1 and p57kip2, which are essential for cell-cell contact inhibition in healthy tissue but are lost or disrupted in cancer cells, primarily due to the loss of E-cadherin in cancer cells (Croix et al., 1998; Cavallaro and Christofori, 2004a; Migita et al., 2008). Therefore, decreased cell-cell adhesion not only increases the potential for metastatic dissemination of cancer cells, but also encourages unchecked cell proliferation through the absence of contact inhibition (Cavallaro and Christofori, 2004b). Indeed, studies has shown a correlation between reduced E-cadherin and *α*-catenin expression with increased tumor cell invasiveness (Zschiesche et al., 1997). Sheng Liu et al. demonstrated that reduced E-cadherin expression was correlated with distant metastasis of GIST and E-cadherin was thus considered as risk factor for GIST metastasis. In our study, E-cadherin had been identified as the top hub gene and to be involved in the process of tumorigenesis and liver metastasis of GIST. The results of our study demonstrated decreased expression levels of E-cadherin were associated with unfavorable OS in gastrointestinal tumors. Therefore, we believe that it mediates the liver metastasis of GIST and can be used as a target for the treatment of metastatic GIST.

ETV1, a transcription factor from the ETS family, is a master regulator of the normal lineage specification and development of the ICCs which are the precursors to GIST (Chi et al., 2010). Hao-Chen Wang et al. reported that upregulating ETV1 expression induced CXCR4 expression, which promoted liver metastasis of GIST (Wang et al., 2016). We compared ETV1 expression in our cohort and found that ETV1 are upregulated in GIST tissues of patients with liver metastasis compared with corresponding non-tumor tissue (Supplementary Figures S2A, C). Our result supports ETV1’s stimulative role in liver metastasis of GIST. Besides, it has been demonstrated that ETV4 expression impacted Wnt/catenin signaling and was correlated to an aggressive phenotype in GIST (Zeng et al., 2017). However, our results showed no significant difference in ETV1 expression levels in GIST compared to the adjacent tissues in both our cohort and GSE13861 (Supplementary Figures S2B, D). Further research in this area is needed.

The major limitation of the present study is that Tumor transcriptome programs are rather diverse, both within tumor cells due to somatic genetic changes and within tumor microenvironments due to extensive infiltration of the stroma and other cell types in the tumor. An average gene expression profile from microarray can mask the real signals causing the liver metastasis of GIST from a rare cell population or cell type. Besides, it has been indicated that long non-coding RNAs (LncRNAs) participate in certain pro-metastatic stages, such as the epithelial mesenchymal transition, invasion and migration, and organotrophic colonization, and they also have an impact on the metastatic tumor microenvironment (Amirnasr et al., 2020; Liu et al., 2021). The gene chips we used in current study only contain probes for protein-coding mRNAs but not LncRNAs. Thus, further researches should be conducted to elucidate the potential function of LncRNAs in liver metastasis of GIST. Moreover, a direct comparison of liver metastases and primary sites of GIST maybe a better study protocol. But, on one hand, liver metastases from GIST patients are difficult to obtain because they are usually treated by ablation. On the other hand, we think that the transcription level of GIST with liver metastasis has already changed before metastasis, the potential role of these genes in promoting liver metastasis cannot be ignored. This information is lost if direct compare liver metastases samples and primary lesions. It would be better if we collected GIST specimens without liver metastasis and adjacent tissues at the same time. This reduces batch effects compared to using data from GEO databases for comparison. Furthermore, there is currently no public database contains both prognostic and gene sequencing data of GIST. And, our cohort contained too few cases (only 18 patients) to survival analysis. So, we can only retreat to the next best, using TCGA database for survival analysis. Whether these hub genes in GIST have prognostic value remains to be further confirmed.

In summary, through analyzing data of self-made whole-genome gene expression profiling and GEO dataset, we identified those signal pathways and hub genes that played significant roles in the tumorigenesis and liver metastasis of GIST. Further studies with larger sample sizes should be carried out to validate the present findings. Additionally, experimental evidence is warranted to investigate the functional roles of the identified hub genes in the liver metastasis of GIST. We sincerely hope that this present study will contribute to the discovery of therapeutic target for liver metastatic GIST.

## Data Availability

The original contributions presented in the study are included in the article/Supplementary Materials, further inquiries can be directed to the corresponding author.

## Supplementary material

SUPPLEMENTARY FIGURE S1 Identification of differentially expressed genes of GSE13861 dataset. **(A)** Volcano map of differentially expressed genes (Upregulated genes in red, downregulated genes in blue). **(B)** Hierarchical clustering heatmap of DEGs screened on the basis of FC >2.0 and a corrected P value <0.05. **(C)** Shows PCA results of our cohort. **(D)** Visual hierarchical cluster analysis.

SUPPLEMENTARY FIGURE S2 Expression of ETV1 and ETV4. **(A, B)** Expression of ETV1 and ETV4 in GIST (with liver metastasis) tissues and correspond non-tumor tissues in our cohort. **(C, D)**. Expression of ETV1 and ETV4 in primary GIST tissues and correspond non-tumor tissues in GSE13861 dataset.
