# Supplementary figures and images for: Transcriptomic study of gastrointestinal stromal tumors with liver metastasis
Source: Front Genet. 2023 Feb 23;14:1007135. doi: 10.3389/fgene.2023.1007135 (PMC9996342; doi:10.3389/fgene.2023.1007135)

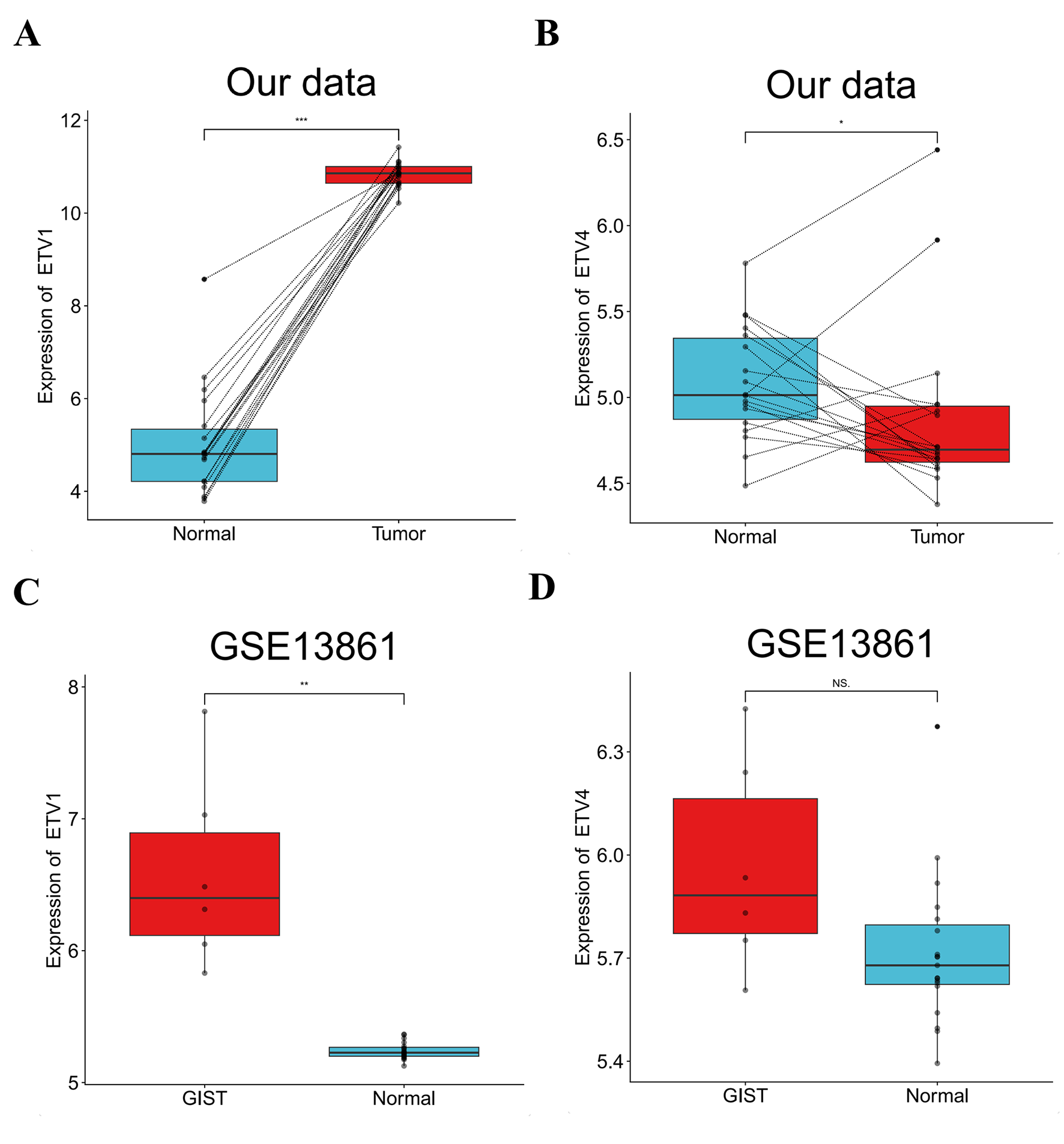

Supplement: Supplementary file 3 [file Image2.tif]

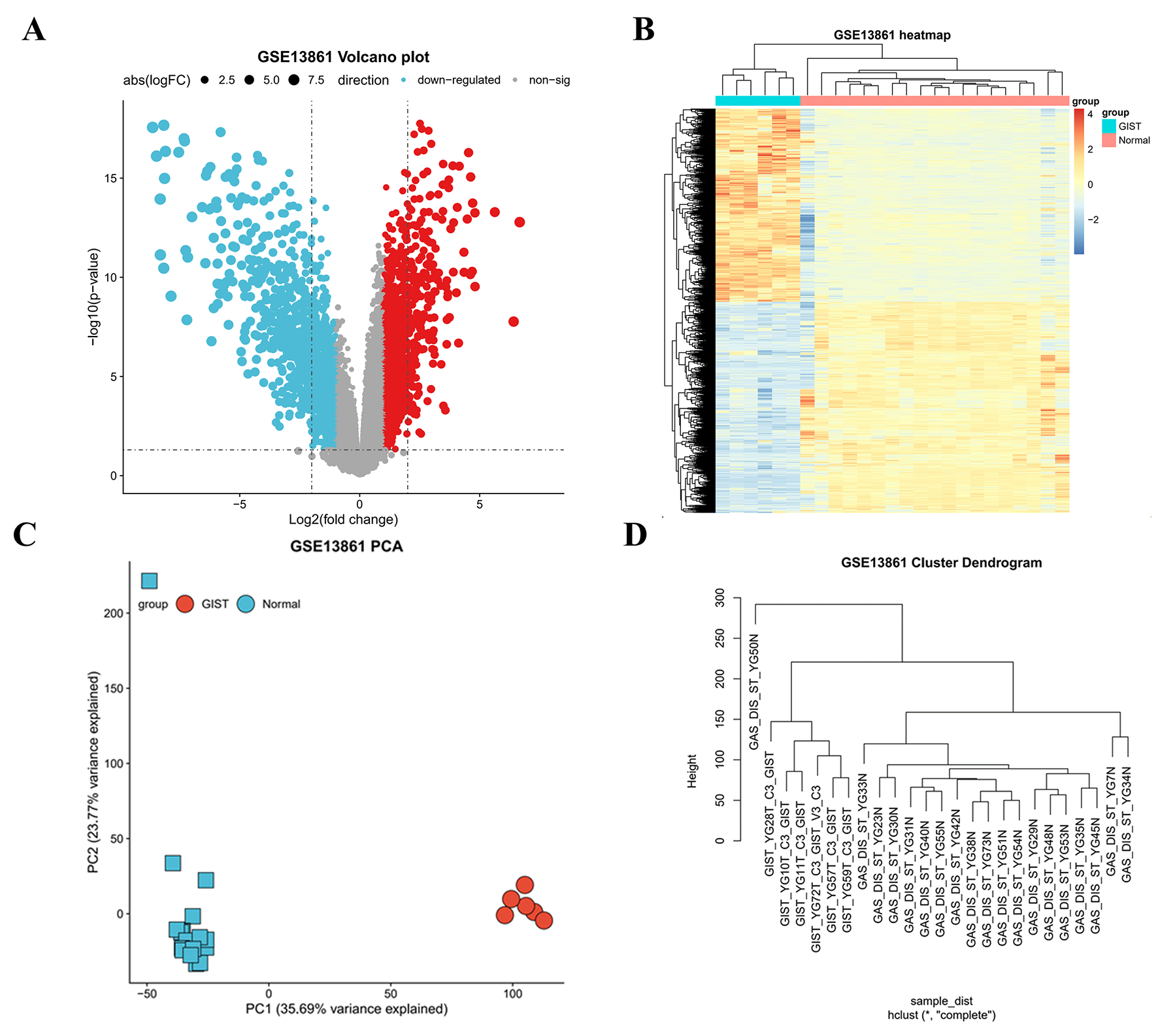

Supplement: Supplementary file 4 [file Image1.tif]
